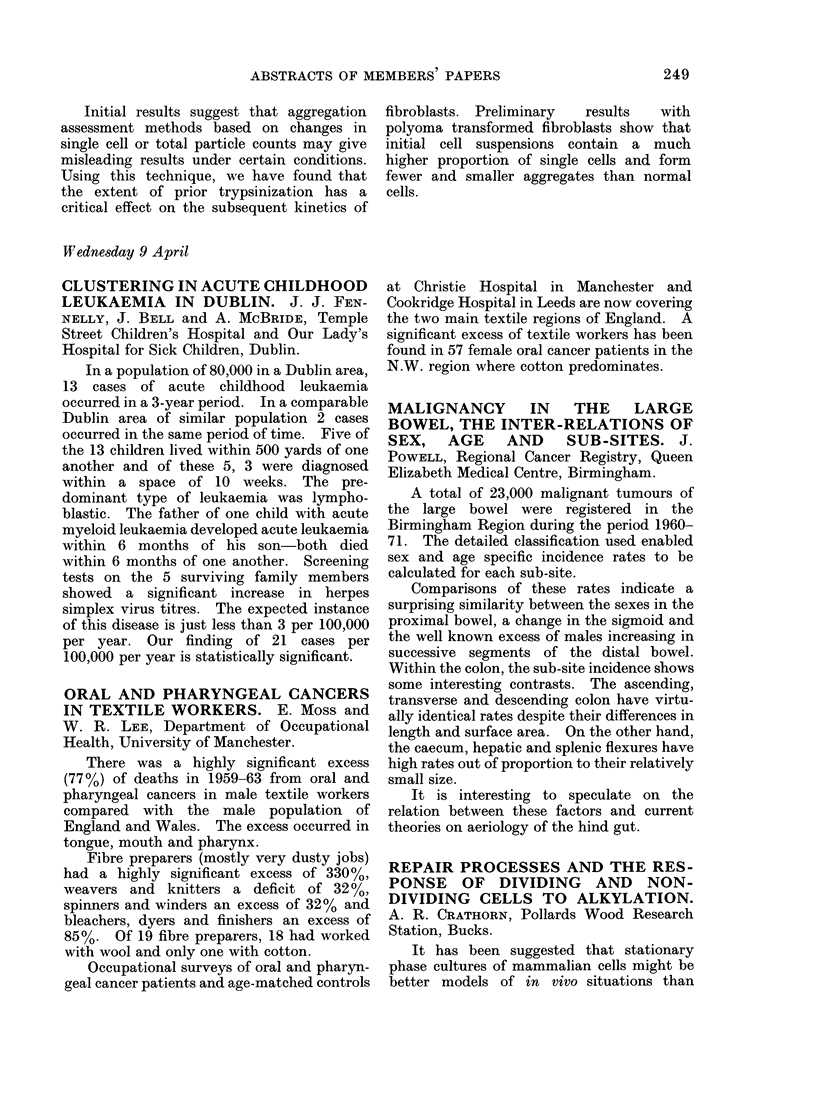# Proceedings: Clustering in acute childhood leukaemia in Dublin.

**DOI:** 10.1038/bjc.1975.184

**Published:** 1975-08

**Authors:** J. J. Fennelly, J. Bell, A. McBride


**CLUSTERING IN ACUTE CHILDHOOD LEUKAEMIA IN DUBLIN.** J. J. Fennelly, J. Bell and A. McBride, Temple Street Children's Hospital and Our Lady's Hospital for Sick Children, Dublin.

In a population of 80,000 in a Dublin area, 13 cases of acute childhood leukaemia occurred in a 3-year period. In a comparable Dublin area of similar population 2 cases occurred in the same period of time. Five of the 13 children lived within 500 yards of one another and of these 5, 3 were diagnosed within a space of 10 weeks. The predominant type of leukaemia was lymphoblastic. The father of one child with acute myeloid leukaemia developed acute leukaemia within 6 months of his son—both died within 6 months of one another. Screening tests on the 5 surviving family members showed a significant increase in herpes simplex virus titres. The expected instance of this disease is just less than 3 per 100,000 per year. Our finding of 21 cases per 100,000 per year is statistically significant.